# The Impact of Corticosteroids on the Outcome of Fungal Disease: a Systematic Review and Meta-analysis

**DOI:** 10.1007/s12281-023-00456-2

**Published:** 2023-02-23

**Authors:** Zhaolun Li, David W. Denning

**Affiliations:** 1 School of Medical Sciences, Faculty of Biology, Medicine and Health, The University of Manchester, Oxford Road, Manchester, M13 9PL UK; 2 Manchester Fungal Infection Group, The University of Manchester, Manchester Academic Health Sciences Centre, Manchester, M23 9LT UK; 3 Manchester Fungal Infection Group, Infectious Diseases in Global Health, CTF Building, Grafton Street, Manchester, M13 9NT UK

**Keywords:** Fungal disease, Prednisolone, Methylprednisolone, Hydrocortisone, Blindness

## Abstract

**Purpose of Review:**

Corticosteroids have a complex relationship with fungal disease — risk for many, benefit for others. This systematic review aims to address the effect of corticosteroids on mortality and visual outcome in different fungal diseases.

**Recent Findings:**

Corticosteroids are a risk factor of aspergillosis for patients who have COVID-19, and they also led to a worse outcome. Similarity, corticosteroids are a risk factor for candidemia and mucormycosis. Some researchers reported that using topical corticosteroid in keratitis was associated with worse visual outcome if fungal keratitis. Some studies showed that corticosteroids are linked to a negative outcome for non-HIV patients with *Pneumocystis jirovecii* pneumonia (PCP), in contrast to those with HIV and PCP.

**Summary:**

In 59 references, we found that corticosteroid therapy showed a worse clinical outcome in invasive aspergillosis (IA) (HR: 2.50, 95%CI: 1.89–3.31, *p* < 0.001) and chronic pulmonary aspergillosis (CPA) (HR: 2.74, 95%CI: 1.48–5.06, *p* = 0.001), PCP without HIV infection (OR: 1.29, 95%CI: 1.09–1.53, *p* = 0.003), invasive candidiasis and candidaemia (OR: 2.13, 95%CI: 1.85–2.46, *p* < 0.001), mucormycosis (OR: 4.19, 95%CI: 1.74–10.05, *p* = 0.001) and early in the course of fungal keratitis (OR: 2.99, 95%CI: 1.14–7.84, *p* = 0.026). There was equivocal outcome in cryptococcal meningoencephalitis in AIDS and primary coccidioidomycosis, while corticosteroid therapy showed a better outcome in PCP in HIV-infected patients (RR: 0.62, 95%CI: 0.46–0.83,* p*=0.001) and fungal keratitis patients after keratoplasty surgery (OR: 0.01, 95%CI: 0.00–0.41, *p* = 0.041) and probably in cryptococcal meningoencephalitis in non-immunocompromised patients. A sub-analysis in invasive aspergillosis and CPA showed that use of more than 2 mg/kg/day of prednisolone equivalents per day is a significant factor in increasing mortality (HR: 2.94, 95%CI: 2.13–4.05, *p* < 0.001). Corticosteroid therapy during invasive fungal disease was usually associated with a slightly or greatly increased mortality or worse visual outcome (in fungal keratitis), with two disease exceptions. Avoiding the addition of corticosteroids, or minimising dose and duration in those who require them, is likely to improve the outcome of most life- and vision-threatening fungal diseases. This review provides a cornerstone for further research in exploring the accuracy of suitable dose and duration of corticosteroids treatment in fungal diseases.

**Supplementary Information:**

The online version contains supplementary material available at 10.1007/s12281-023-00456-2.

## Introduction

More than 1 billion people are affected by fungal infections (mycosis) each year. Well over 2 million infections are life-threatening, especially in immunocompromised patients [1], for example individuals with solid organ or stem cell transplants. This population has expanded due to medical advancements, increasing the incidence of significant fungal infections [2]. Immunosuppression as a risk factor highlights the crucial function of the immune system in controlling opportunistic fungal infections. In order to combat this, increasing host immune function or targeting interactions between the host immune system and fungus might be used in conjunction with antifungal medications [3].

Corticosteroids are commonly used in immunocompromised patients. They are a risk factor for development of invasive fungal infection (IFI) [4] and chronic pulmonary aspergillosis (CPA) in patients with pulmonary nontuberculous mycobacteria [5, 6]. Several studies reported that the early use of corticosteroids for acute graft-versus-host disease is a key risk factor for IA [7, 8, 9, 10]. Additionally, topical steroid is regarded a major risk factor for the development of fungal keratitis [11]. Recently, many studies reported that corticosteroids were the risk factor for COVID-19-associated pulmonary aspergillosis (CAPA) [12, 13, 14, 15]. Corticosteroids limit vasodilation, boost capillary permeability (humoral response) and leukocyte migration to wounded tissue (cellular response). They exert most of their immunosuppressive and anti-inflammatory effects via the glucocorticoid receptor, which inhibits the activity of critical transcriptional regulators of pro-inflammatory genes, including NF-κB, in leukocytes. In addition, corticosteroids decrease the number of monocytes and macrophages in circulation by inhibiting their myelopoiesis and bone marrow release. Notably, glucocorticoids suppress phagolysosomal fusion in macrophages by stabilising lysosomal membranes during phagocytosis. Due to the inhibitory effect on phagocytic function, there is an immediate danger of infection with high-dose glucocorticoid therapy [16], especially in HSCT recipients and patients with autoimmune diseases such as systemic lupus erythematosus.

Nowadays, corticosteroids may be necessary to control an underlying disease even when an IFI occurs. It is usually a major clinical dilemma as to whether to and to what extent to withdraw their use. Furthermore, corticosteroids are advocated for some fungal infections as adjunctive therapy, notably PCP in AIDS patients. No comprehensive systematic review has summarised the outcome of the corticosteroids in patients with fungal disease except a meta-review of PCP [17], which only focused on randomised controlled trials. Our systematic review assesses fungal disease patients’ outcomes (survival, vision) with corticosteroid treatment.

## Methods

The Preferred Reporting Items for Systematic Reviews and Meta-Analyses (PRISMA) guidelines were followed throughout the whole process of conducting and presenting this systematic review. This study did not require ethical approval or informed consent because it was a systematic review of previously published studies. The study was registered in INPLASY (INPLASY202280110) in August 2022.

### Search Strategy and Study Selection

A comprehensive literature search was performed through PubMed, Web of Science, Embase and CNKI databases using the following keywords, title/abstracts and Medical Subject Headings (MeSH) terms: the name of fungal disease (aspergillosis, candidiasis, *Pneumocystis jirovecii* pneumonia, cryptococcal meningoencephalitis, fungal keratitis, fusariosis, mucormycosis, allergic fungal rhinosinusitis, *Talaromyces*, dimorphic fungal disease) AND (corticosteroids or glucocorticoid or steroids) AND (outcome or survival or vision or organ transplant loss), for articles published from inception to June 1st, 2022, in peer-reviewed journals. We searched each fungal disease separately rather than combining them by “OR” as we tried to search more specifically. Studies published in languages other than English and Chinese were excluded if no translated version of the manuscript was available. In addition, we performed a search manually for other reviews (either systematic or narrative). EndNote^tm^20 was utilised to manage the bibliography received from the search during the entire review procedure. One author screened the search results based on the title and abstract independently and another author helped to confirm eligibility based on the inclusion criteria.

### Inclusion Criteria and Exclusion Criteria

Studies were included that met the following criteria: (1) original large observational case series or randomised controlled studies; (2) reports of outcome (survival, organ transplant loss or vision change) are clearly described; (3) reports with data on outcome between patients treated with and without corticosteroids; (4) the diagnostic criteria for fungal disease were clearly provided and internationally accepted. Information was also extracted on corticosteroid dose and duration related to outcome, if available, and analysed.

The exclusion criteria were as follows: (1) no information on patient outcome; (2) outcome not related to therapy of corticosteroid; (3) in vitro and experimental animal studies; (4) reports of single case experiences or small series.

### Data Extraction and Quality Assessment

One author (ZL) extracted all relevant data on the main characteristics (author; published year; study country and period; design and type of fungal disease, study population) and results (outcome and hazard ratios (HR); odds ratio (OR) risk ratios (RR)) of the selected studies. Additionally, dose analysis (> 2 mg/day) in aspergillosis of corticosteroid was defined as prednisone or an equivalent total dose of another corticosteroid. The extracted data from full texts of included studies was added into a standardised Excel (Microsoft Corporation) form.

Quality assessments used the Newcastle–Ottawa Scale (NOS) for cohort and case control studies and version 2 of the Cochrane risk-of-bias tool for randomised controlled trials (RCTs) [18]. The total NOS score ranges from 0 to 9 [19]. The quality of a study is given a score of 0–3 for low quality, 4–6 for moderate quality and 7–9 for high quality. During the evaluation of each study, any uncertainties were discussed with the other author. Cochrane risk-of-the bias instrument consists of seven bias domains: sequence generation and allocation concealment, blinding of participants and employees, blinding of outcome assessors, incomplete outcome data, selective reporting and additional bias. For each bias category, the instrument invites users to rate the risk of bias as “high”, “low” or “unclear” and to provide justification for their judgments [20]. We also performed a series of quality assessments of cohort studies (Table S1a–S1h). The quality of assessments of RCTs is combined with the forest plot (Fig. 2).

### Data Analysis

The review includes papers and studies using different study designs, so combining and analysing the data was difficult. We performed a meta-analysis of RCTs and cohort and case control studies separately. The software of Revman 15 was used for RCTs while the STATA 17 was used for cohort studies. We assessed heterogeneity between studies using the I^2^ statistic. Additionally, if RR and OR were not shown in the original paper, we calculated them using the original data extracted from each paper. For RCTs, we used the data of events and the total patients’ number in both treatment and control groups. For cohort studies and case control studies, we combined the OR and HR in STATA to perform a meta-analysis, given that these cohorts recruited groups of patients with a similar risk for death, a single episode of infection and that most deaths occurred within 30–45 days. Moreover, we extracted *p* values from STATA 17 and added them manually on the forest plot as the *p* value does not show in the figure when using the STATA 17. We also calculated 95% confidence intervals (CIs) in STATA. Data are displayed using forest plots and the test of the overall effect, *p* value and RR reflect the results. Moreover, subgroup analyses were performed based on the dose of corticosteroid in aspergillosis and disease types in candidiasis.

## Results

**Fig. 1:**
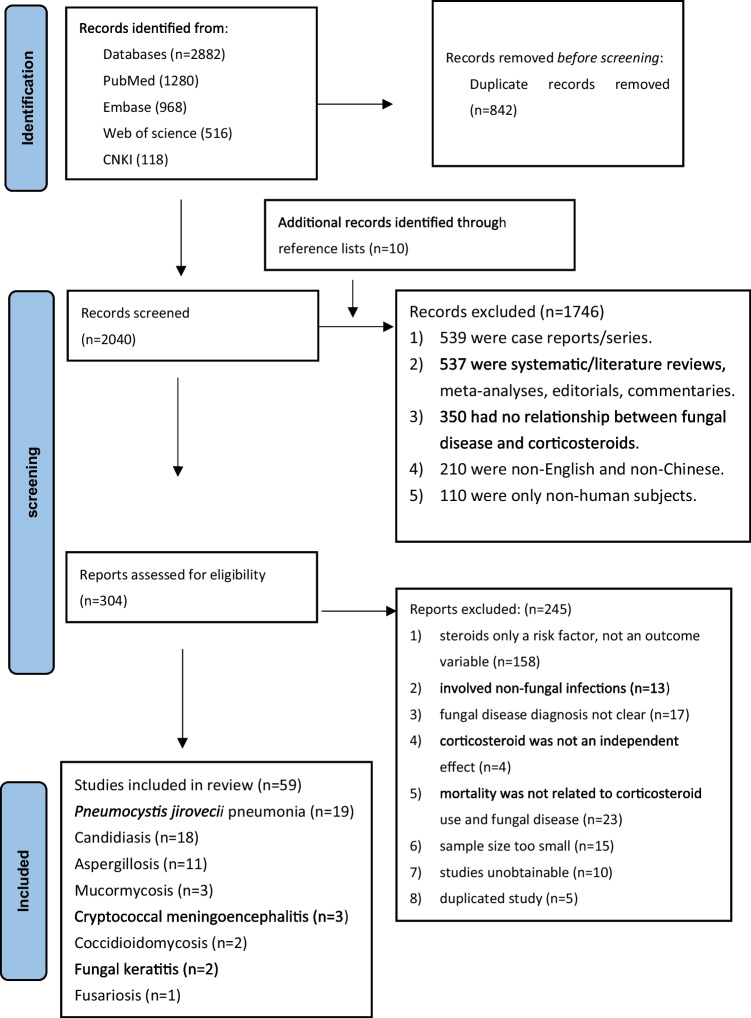
Flow diagram of study selection. Fungal disease with corticosteroid therapy

In the initial search, 2882 studies were identified. After removing duplicates and papers not meeting the inclusion criteria, 59 studies satisfied the inclusion criteria and were included in this meta-analysis (Fig. 1). These studies concerned different fungal diseases including 19 PCP studies, 18 of invasive candidiasis, hepatosplenic candidiasis and candidemia, 11 of invasive aspergillosis and CPA, three of mucormycosis, three of cryptococcal meningoencephalitis, two of fungal keratitis, two of coccidioidomycosis and one of fusariosis. A summary of these studies is provided followed by a meta-analysis.

### *Pneumocystis jirovecii* Pneumonia

Treatment with corticosteroid demonstrated an opposite result in PCP patients with or without HIV. In HIV patients, the overall risk of death was reduced to 63% with corticosteroids, but in those with other risk factors, was increased by 29%. This section includes 19 studies (11 cohort studies, 7 RCTs and one case–control study) and which patient group was included is displayed in Table 1.

**Table 1:** Characteristics of the studies — *Pneumocystis jirovecii* pneumonia (PCP)

Study	Study period (years/months)	Country	Study design	Sample size	Age (years)	Host group
Clement, 1989 [21]	NA	NA	RCT	NA	NA	HIV
Montaner, 1990 [22]	NA	NA	RCT	37	NA	HIV
Bozzette, 1990 [23]	1987–1989, 3 years	NA	RCT	251	36	HIV
Gagnon, 1990 [24]	1989–1990, 2 years	USA	RCT	23	38	HIV
Nielsen, 1992 [25]	1988–1990, 3 years	Europe	RCT	59	37.1	HIV
Walmsley, 1995 [26]	NA	NA	RCT	78	NA	HIV
Pareja, 1998 [27]	1989–1995, 7 years	USA	Case control retrospective	30	59	Non-HIV
Delclaux, 1999 [28]	1988–1996, 9 years	France	Cohort retrospective	31	49	Non-HIV
Pagano, 2002 [29]	1990–1999, 10 years	Italy	Cohort retrospective	55	47	Non-HIV
Zahar, 2002 [30]	1989–1999, 10 years	France	Cohort retrospective	39	52	Non-HIV
Roblot, 2003 [31]	1995–1999, 5 years	France	Cohort retrospective	60	59	Non-HIV
Bolle´e, 2007 [32]	2001–2006, 6 years	France	Cohort retrospective	56	49	Non-HIV
Terblanche, 2008 [33]	2005–2006 2 years	South Africa	RCT	100	3.3 months	Non-HIV
Moon, 2011 [34]	2007–2010, 4 years	Korea	Cohort retrospective	88	56.5	Non-HIV
Kofteridis, 2014 [35]	2004–2013, 10 years	Greece	Cohort retrospective	62	65.2	Non-HIV
Wieruszewski, 2018 [36]	2006–2016, 11 years	USA	Cohort retrospective	323	65	Non-HIV
Liu, 2019 [37]	2015–2016, 2 years	China	Cohort retrospective	84	NA	Non-HIV
Inoue, 2019 [38••]	2010–2016, 6 years	Japan	Cohort retrospective	1299	67.5	Non-HIV
Assal, 2021 [39]	2010–2017, 8 years	France	Cohort retrospective	133	64.9	Non-HIV

*RCT*, randomised controlled study; *HIV*, human immunodeficiency virus

**Fig. 2:**
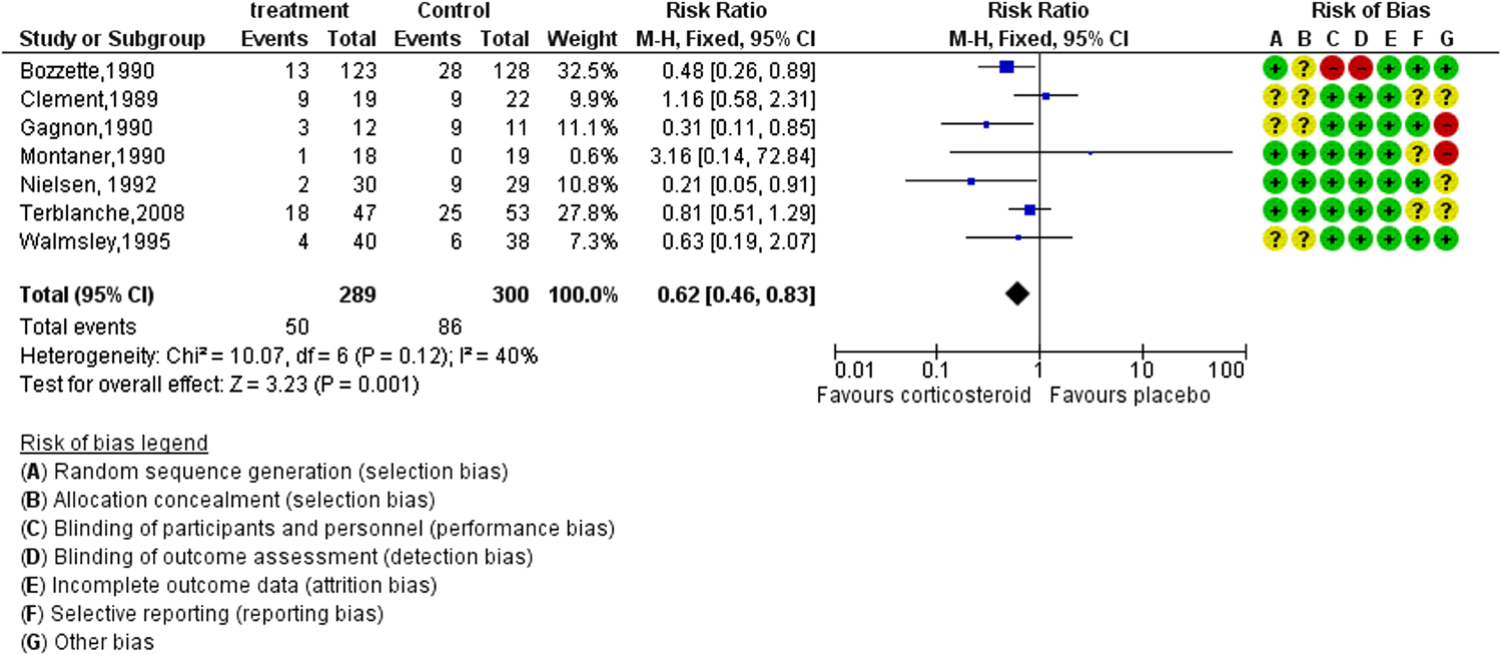
Pooled risk ratios for *Pneumocystis jirovecii* pneumonia in HIV-infected patients

Most RCT studies showed that treatment with corticosteroids had a better outcome for PCP patients with HIV. The focus of these studies was patients with moderate or severe PCP. Corticosteroid regimens differed in dose and duration. Although Clement et al. [21] and Montaner et al. [22] found that patients in the corticosteroid group had 1.16-fold and 3.16-fold higher risk of mortality than patients in the placebo group, respectively, the weight of these two studies was only about 10% (Fig. 2). Consequently, the pooled analysis of 7 RCTs with 589 participants with HIV showed a reduced mortality rate in the corticosteroid group (RR: 0.62, 95%CI: 0.46–0.83, *p *= 0.001 *I*^2 ^= 40%).

Only one study addressed childhood fatality related to PCP and corticosteroids [33]. In infants less than 18 months old diagnosed with PCP, those given prednisone had a 43% better chance of survival (HR: 0.57, 95%CI: 0.30–1.07, *p* = 0.08) which was not statistically significant.

**Fig. 3:**
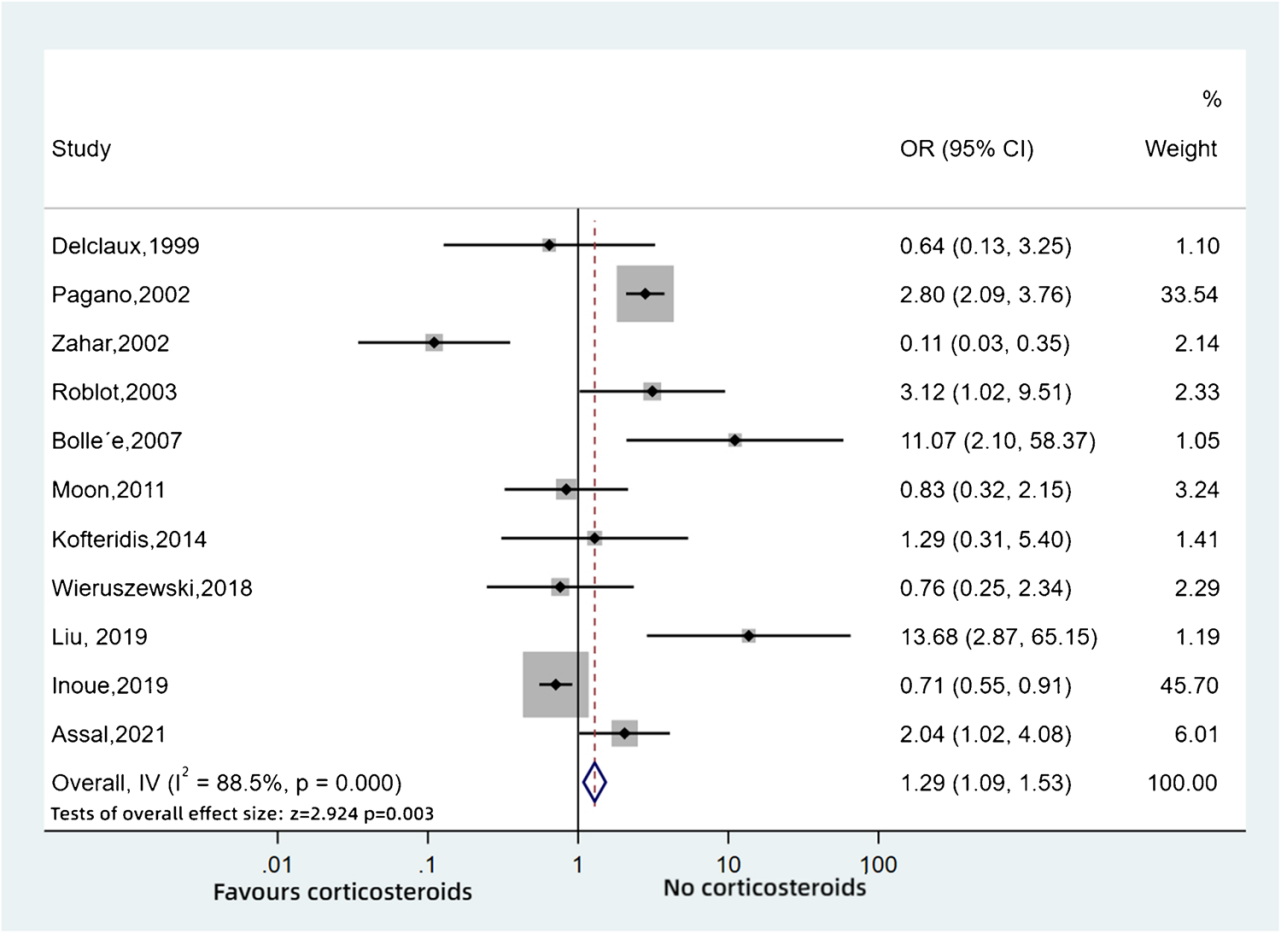
Pooled odds ratios for PCP in patients without HIV infection

By contrast, the 11 retrospective cohort studies included 2230 patients without HIV infection, with some conflicting results (Fig. 3). Pagano [29] found that patients who received corticosteroids had an increased risk of death, while Inoue [38••] showed that steroids as adjunctive therapy significantly improved survival. Additionally, there was no significant difference in PCP patients with or without steroids among Moon’s [34], Kofteridis’s [35] and Wieruszewski’s studies [36]. However, the overall result indicates that treatment with steroids in non-HIV PCP patients had a worse outcome (OR: 1.29, 95%CI: 1.09–1.53, *p* = 0.003, *I*^2^ = 88.5%).

Only one case–control study [27] addressed the dose of steroid, which showed that in-hospital mortality rates were similar for the two comparison groups, including 44% (7/16) for the enhanced high-dose steroid group and 36% (5/14) for the low-dose steroid group non-HIV (*p* = 0.722).

### Aspergillosis

**Fig. 4:**
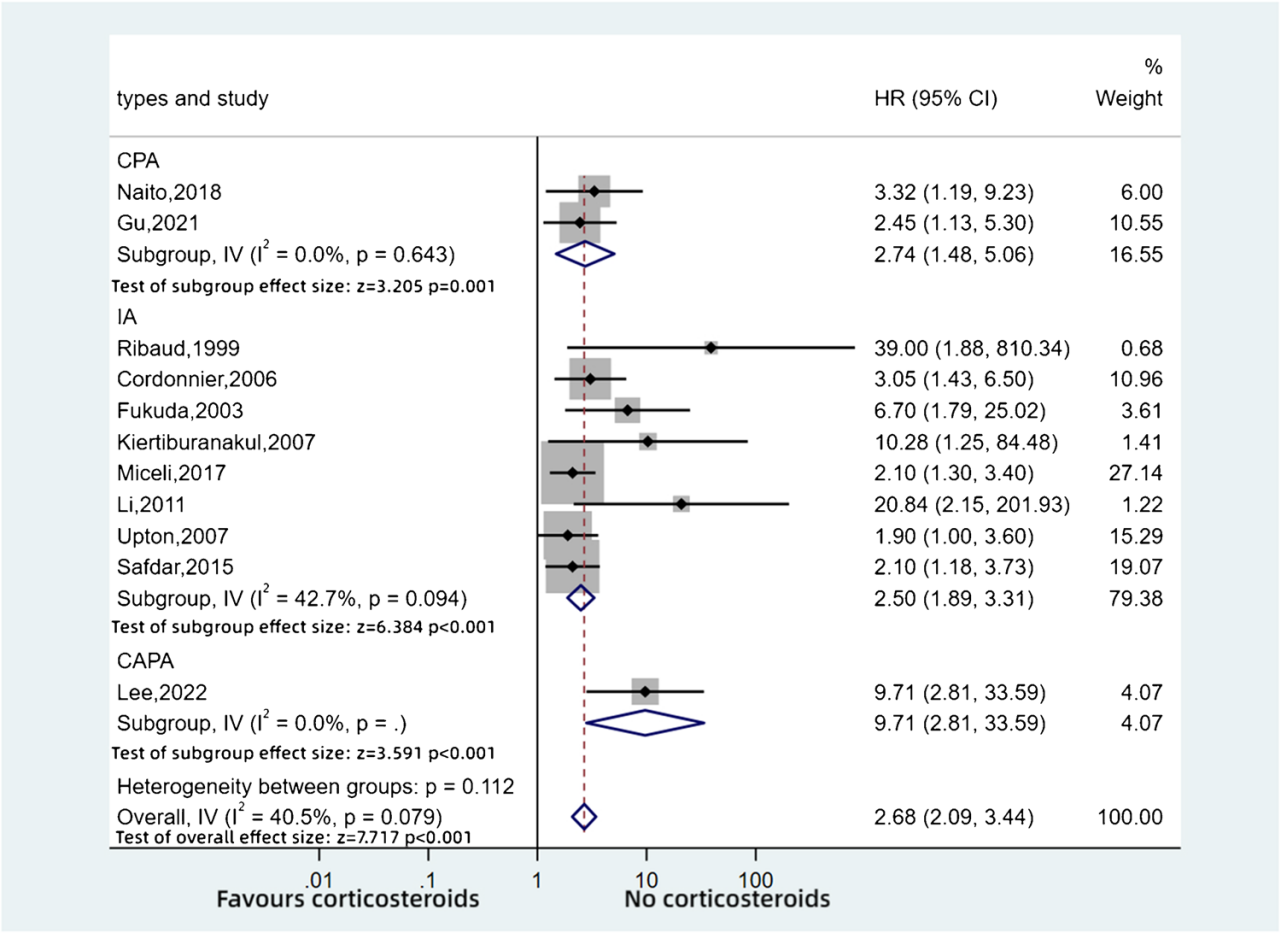
Pooled hazard ratios for aspergillosis

Eleven cohort studies reported the mortality of aspergillosis in patients who received adjunctive steroid therapy, often perceived to be necessary to prevent transplant rejection or to control other underlying disease. This analysis includes patients with invasive aspergillosis (IA) (8 studies, 944 patients), CPA (2 studies, 122 patients) and CAPA (1 study, 218 patients). As for CPA, one study [40] reported that an accumulated total dose of > 700 mg prednisolone equivalents had a worse outcome for patients (HR: 2.45, 95%CI: 1.13–5.30, *p* = 0.023), while another study [41] also reported that use of corticosteroids was associated with increased mortality (HR: 3.32, 95%CI: 1.23–9.51, *p* = 0.00177). Additionally, one study [42••] of CAPA revealed that daily use of high-dose corticosteroids predicted CAPA and correlated with higher death rates (HR: 9.71, 95%CI: 2.81–33.59). A total of 1284 patients were included in these studies (Table 2). All studies showed that steroid therapy was a risk factor for death. The meta-analysis demonstrated that the patients who received steroids had a 2.71-fold higher risk of death than those without steroid treatment (HR: 2.68, 95%CI: 2.09–3.44, *p* < 0.001, *I*^2^ = 40.5%) (Fig. 4).

**Table 2:** Characteristics of the studies — invasive aspergillosis, CPA and CAPA

Study	Study period (years/months)	Country	Study design	Sample size	Age (years)	Types of disease
Ribaud, 1999 [43]	1994, 10 months	French	Cohort retrospective	27	31	IA
Fukuda, 2003 [44]	1997–2001, 4 years	USA	Cohort retrospective	25	NA	IA
Cordonnier, 2006 [45]	2002, 1 year	French	Cohort retrospective	51	40.1	IA
Kiertiburanakul, 2007 [46]	2000–2005, 5 years	Thailand	Cohort retrospective	94	47.9	IA
Upton, 2007 [47]	1990–2004, 15 years	USA	Cohort retrospective	405	42.2	IA
Li, 2012 [48]	2000–2007, 7 years	China	Cohort retrospective	190	NA	IA
Safdar, 2015 [49]	2002–2006, 5 years	USA	Cohort retrospective	91	48	IA
Miceli, 2017 [50]	2007–2012, 6 years	USA	Cohort retrospective	61	56	IA
Naito, 2018 [41]	2010–2015, 5 years	Japan	Cohort retrospective	62	69.5	CPA
Gu, 2021 [40]	2014–2019, 5 years	China	Cohort retrospective	60	71.5	CPA
Lee, 2022 [42••]	2020–2021, 1 year	Korea	Cohort retrospective	218	62	CAPA

*IA*, invasive aspergillosis; *CPA*, chronic pulmonary aspergillosis; *CAPA*, COVID-19-associated pulmonary aspergillosis

**Fig. 5:**
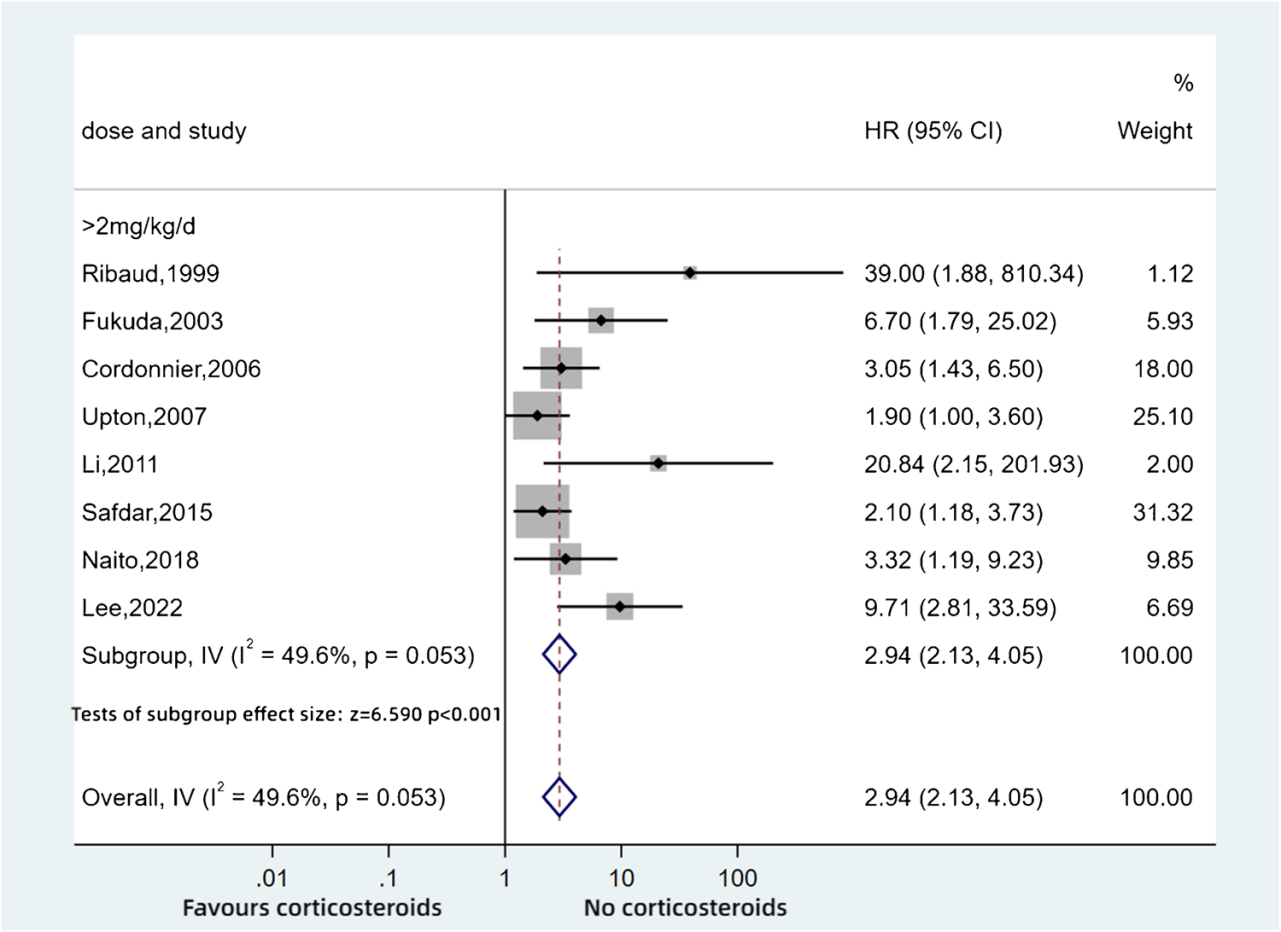
Pooled hazard ratios for aspergillosis

We also undertook a subgroup analysis based on the daily dose of steroids in CPA and IA patients combined (Fig. 5). We found that patients who received > 2 mg/kg/day prednisolone equivalents showed a higher mortality than those on lower doses, which was significantly different (HR: 2.94, 95%CI: 2.13–4.05, *p* < 0.001).

### Candidiasis and Candidemia

**Fig. 6:**
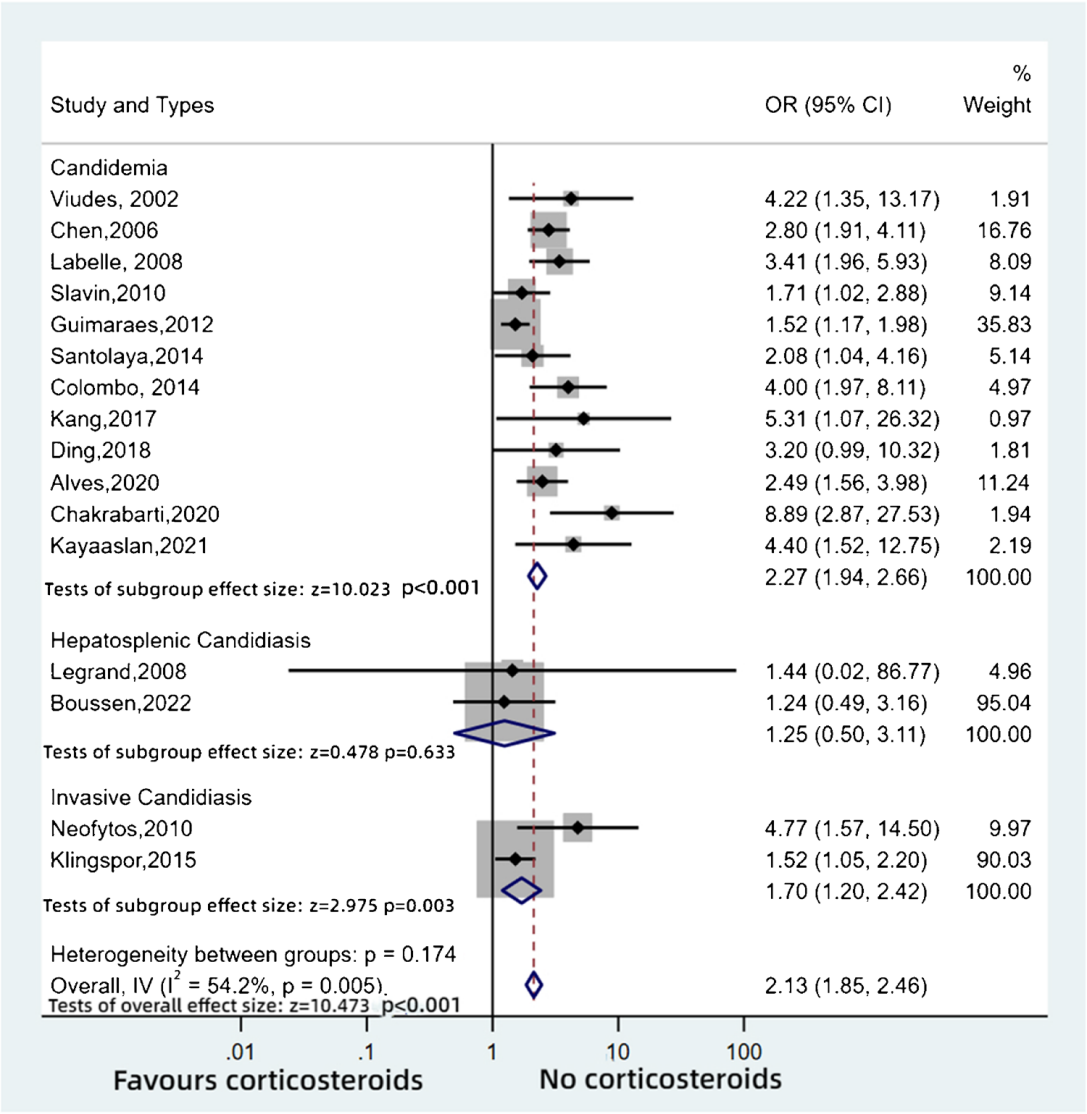
Pooled odds ratios for invasive candidiasis and candidemia

In total, 19 studies reported outcomes in various disease entities caused by *Candida* spp. related to corticosteroid use: 18 cohort studies and one case–control study (Table 3). The pooled analysis of 16 cohort studies with 6607 participants had a higher risk of death with exposure to corticosteroids (OR: 2.13, 95%CI: 1.85–2.46, *p* < 0.001, *I*^2^ = 54.2) (Fig. 6). Subgroup analysis was based on the disease types, including candidemia, hepatosplenic (chronic disseminated) candidiasis and invasive candidiasis. Patients who had candidemia and invasive candidiasis with steroid therapy demonstrated a significantly higher risk of death than those who did not receive steroids (OR: 2.27, 95%CI: 1.94–2.66, *p* < 0.001; OR: 1.70, 95%CI: 1.20–2.42, *p* = 0.003, respectively).

**Table 3:** Characteristics of the included studies — invasive candidiasis and candidemia

Study	Study period (years/months)	Country	Study design	Sample size	Age (years)	Types of disease
Viudes, 2002 [51]	1995–1997, 3 years	Spain	Cohort retrospective	145	NA	Candidemia
Chen, 2006 [52]	2001–2004, 5 years	Australia	Cohort prospective	857	56	Candidemia
Labelle, 2008 [53]	2004–2006, 2.5 years	USA	Cohort prospective	245	57.5	Candidemia
Legrand, 2008 [54]	1991–2004, 14 years	France	Cohort retrospective	10	18.6	Hepatosplenic candidiasis
Neofytos, 2010 [55]	2001–2004, 3 years	USA	Cohort retrospective	429	60.5	Invasive candidiasis
Slavin, 2010 [56]	2004–2007, 3.5 years	Australia	Cohort prospective	288	53.8	Candidemia
Munoz, 2011 [57]	1985–2008, 24 years	Spain	Case control study	59	67	Candida tropicalis fungaemia
Guimaraes,2012 [58]	1994–2004, 10.5 years	Brazil	Cohort retrospective	987	72	Candidemia
Santolaya, 2014 [59]	2008–2010, 2 years	Latin America	Cohort prospective	213	2	Candidemia
Colombo, 2014 [60]	2008–2012, 9 years	Brazil	Cohort prospective	1392	62	Candidemia
Klingspor, 2015 [61]	2006–2008, 3 years	Europe	Cohort prospective	779	63	Invasive candidiasis
Kang, 2017 [62]	2007–2014, 8 years	Korea	Cohort retrospective	72	66	Candidemia
Ding, 2018 [63]	2010–2015, 5.5 years	China	Cohort retrospective	72	62.5	Candidemia
Jang, 2018 [64]	2013–2016, 4 years	Korea	Cohort retrospective	21	51	Hepatosplenic candidiasis
Alves, 2020 [65]	2009–2016 8 years	Brazil	Cohort retrospective	335	NA	Candidemia
Chakrabarti, 2020 [66]	2011–2012, 1.5 years	India	Cohort prospective in premature neonates and children	487	Neonates < 37 gestation; 28 days to 18 years	Candidemia
Kayaaslan, 2021 [67•]	2019–2021, 2 years	Turkey	Cohort prospective	236	72	Candidemia
Korulmaz, 2021 [68]	2014–2018, 2 years	Turkey	Cohort prospective	85	NA	Invasive candidiasis
Boussen, 2022 [69]	2008–2020, 3 years	France	Cohort retrospective	60	44.5	Hepatosplenic candidiasis

This difference was not apparent in patients with hepatosplenic candidiasis, with no significant difference between the steroid and non-steroid treatments (OR: 1.25, 95%CI: 0.50–3.11, *p* = 0.633), although only 70 patients were included. One cohort study [64] likewise demonstrated no statistically significant differences in mortality in those with hepatosplenic candidiasis receiving corticosteroid medication and those who did not, but the study did not report the original data. Similarity, the study from Turkey [68] also did not report the original data.

A single case–control study [57] concerned *Candida tropicalis* fungaemia. This study of 59 patients reported that patients treated with steroids had an 8.2-fold higher risk of death (OR: 8.2, 95%CI: 1.1–61.9, *p* = 0.04).

### Mucormycosis

**Fig. 7:**
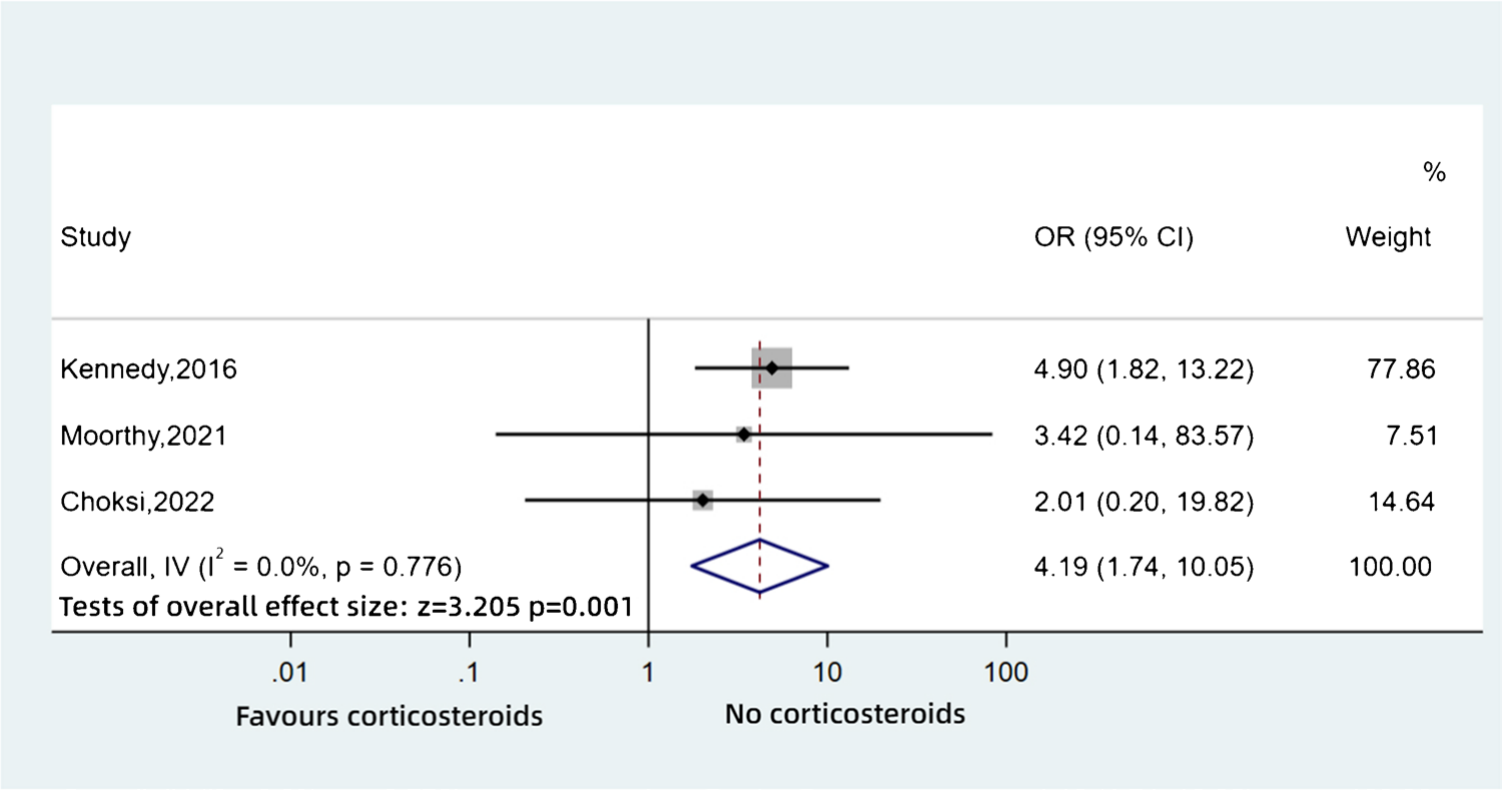
Pooled odds ratios for mucormycosis

Mucormycosis is the second most common invasive mould infection, affecting those with different underlying diseases. India has recently had an epidemic of COVID-19-associated rhino-orbitocerebral mucormycosis (CAM) [70]. Corticosteroids reduce mortality in severe COVID-19 and usage became more common, and as a result, it may be a risk factor for worse outcomes. We found three studies reporting the impact of corticosteroids on survival (Table 4). The pooled analysis of the 164 patients demonstrated that patients who were prescribed steroids had a 4.19-fold higher risk of death than those who did not (OR: 4.19, 95%CI: 1.74–10.05, *p* = 0.001, *I*^2^ = 0%) (Fig. 7). Moorthy et al. [71] also reported on visual outcome, as the orbit of the eye may be involved in mucormycosis. They found loss of vision was more likely in patients treated with corticosteroids (11/16 (69%) versus 1/2 (50%) but was not significant (OR: 2.2, 95%CI: 0.11–42.74, *p* = 0.602) possibly because the study size was too small.

**Table 4:** Characteristics of the included studies — mucormycosis

Study	Study period (years/months)	Country	Study design	Sample size	Age (years)	Types of disease
Kennedy, 2016 [72]	2004–2012, 9 years	Australia	Cohort retrospective	73	54	Haematological malignancy (48.6%), diabetes mellitus (27%), others (24.4%)
Moorthy, 2021 [71]	2020–2020, 7 months	India	Cohort retrospective	18	54.6	Patients with COVID-19 (100%), patients with diabetes (89%)
Choksi, 2022 [70]	2020–2020,3 months	India	Cohort retrospective	73	53.5	Patients with COVID-19 (100%)

### Cryptococcal Meningoencephalitis

The three studies of cryptococcal meningoencephalitis included two cohort studies and one RCT (Table 5). Although these studies reported the relationship between corticosteroid treatment and mortality, one study focused on immunocompetent patients — the others on cryptococcal meningoencephalitis in patients with HIV and post-infectious inflammatory syndrome (PIIRS), respectively. Therefore, we will describe them separately.

**Table 5:** Characteristics of the studies — cryptococcal meningoencephalitis

Study	Study period (years/months)	Country	Study design	Sample size	Age (years)	Types of disease
Seaton, 1997 [73]	1991–1995, 5 years	Papua New Guinea	Cohort retrospective	26	20	Non-immunocompromised
Beardsley, 2016 [74]	2013–2, 2014–8, 1.5 years	Africa, Asia	RCT	450	35	HIV-infected
Anjum, 2021 [75]	2015–2020, 6 years	USA	Cohort prospective	15	51	PIIRS

*HIV*, human immunodeficiency virus; *PIIRS*, post-infectious inflammatory syndrome

Seaton et al. [73] found in their cohort study that blindness was significantly reduced in immunocompetent patients with corticosteroid treatment (1/16 (6.3%) in the steroid group vs 5/10 (50%) in the non-steroid treated group; OR: 0.067, 95%CI: 0.006–0.716, *p* = 0.018).

In a recent study of 15 previously well (non-immunocompromised) patients with ongoing severe symptoms 3–9 weeks after antifungal therapy for cryptococcal meningitis, Anjum et al. [70] found that pulse corticosteroid therapy improved clinical status. Mental status (*p* < 0.0003), headache, visual (*p* < 0.0005) and hearing deficits all improved, along with CSF parameters (*p* < 0.003) and retinal nerve fibre layer thickness (*p* = 0.004). The authors label this entity post-infectious inflammatory response syndrome (PIIRS), which is well supported by detailed immunological investigation.

In Beardsley’s [74] RCT in patients with HIV-associated cryptococcal meningitis, corticosteroids did not reduce mortality (47% in steroids group vs 41% in control group by 10 weeks and 57% vs 49% by 6 months, respectively) among (HR: 1.15, 95%CI: 0.93–1.42, *p* = 0.19; HR: 1.18, 95%CI: 0.99–1.41, *p* = 0.06, *I*^2^ = 0%). However, the study was stopped after enrolling 451 participants on futility and safety grounds.

### Fungal Keratitis

The diagnosis of fungal keratitis is often delayed, and alternative diagnoses are initially considered, for which topical corticosteroid drops are used. We identified two studies describing the relationship between steroids and outcomes of infection (Table 6). Cho’s recent study [76] in 83 patients found that the depth of fungal hyphae infiltration into the cornea and treatment failure including visual acuity was worse and the need for surgical intervention higher in those given topical steroids (OR: 2.99, 95%CI: 1.14–7.84, *p* = 0.026). Corticosteroids appear to alter the pattern of fungal growth, as judged by histology, with more vertical penetration of the cornea [77].

**Table 6:** Characteristics of the studies — fungal keratitis

Study	Study period (years/months)	Country	Study design	Sample size	Age (years)	Reason
Wang, 2016 [78]	2009–2014, 5 years	China	Cohort prospective	244	NA	Corneal graft rejection
Cho, 2019 [76]	2000–2016, 7 years	Korea	Case control	83	62.4	Diagnostic uncertainty

Some patients with fungal keratitis require corneal grafting to preserve vision and prevent globe perforation. This carries a risk of rejection. One of the studies reported that they used steroids only after surgery to prevent rejection of the corneal graft. The study [78] reported that treatment failure was significantly lower in the corticosteroid-treated group (OR: 0.01, 95%CI: 0.00–0.41, *p* = 0.041), and the rate of recurrent fungal keratitis was low.

### Coccidioidomycosis

Two long-term cohort studies were included in our review (Table 7). Blair’s 16-year study [79] included 55 immunocompromised patients with haematologic malignancy and predominantly pulmonary coccidioidomycosis (43/55, 78%). The study reported that 34 patients used steroids and 16 patients died, but they did not give specific data showing how many patients died in the steroid-treated group. In a meta-analysis, they found the usage of steroids was related to increased death along with the status of the hematologic malignancy (*p* = 0.02). By contrast, Azadeh et al. [80] reported 74 patients with primary pulmonary coccidioidomycosis. They found that steroid therapy may help to resolve the symptoms faster than the control group in immunocompetent patients with coccidioidomycosis (a mean of 19 weeks vs 32.3 weeks), but it was not significant (*p* = 0.38).

**Table 7:** Characteristics of the studies — coccidioidomycosis

Study	Study period (years/months)	Country	Study design	Sample size	Age (years)	Types of disease
Blair, 2005 [79]	1987–2002, 16 years	USA	Cohort retrospective	55	66	Mixed, some immunocompetent
Azadeh, 2013 [80]	2005–2011, 7 years	NA	Cohort retrospective	74	NA	Immunocompetent

### Fusariosis

Nucci et al. [81] analysed 84 cases of fusariosis from Brazil and the USA in patients with hematologic malignancy and a mean age of 31.5 years. They found that the actuarial survival rate after recovery from neutropenia was 67% versus 30% for patients who received corticosteroids (HR: 2.18, 95%CI: 1.98–3.96, *p* = 0.001). This finding held up in multivariate analysis, and persistent neutropenia and corticosteroid were additive. It was independently found in those who underwent allogeneic stem cell transplants (HR 12.05, 95%CI: 1.47–100, *p *= 0.02).

### Publication Bias

We assessed the publication bias for cohort studies of PCP, candidasis and aspergillosis. This assessment is only meaningful when using Egger’s regression or funnel plots to analyse in meta-analysis if there are at least 10 studies [82]. The results showed that no publication bias was seen for PCP and candidiasis in supplementary data (S1 and S2), but the probability of publication bias may hamper interpretation in aspergillosis (S3).

## Discussion

This systematic review and meta-analysis is the first systematic evaluation of the outcome of serious fungal disease with corticosteroid treatment. Our review includes various fungal infections in various countries, including randomised controlled trials and observational studies. Most of the research in our analysis pool are retrospective cohort studies as corticosteroid treatment has been confirmed as the risk factor for many fungal infections, so planning and executing RCTs on patients with or without corticosteroid to possibly demonstrate a worse outcome would be unethical in most circumstances.

We analysed patients’ outcome (mortality and vision change) linked to the effect of corticosteroid treatment as a discrete analysis. Previously corticosteroids have been assessed as a risk factor for development of a serious fungal disease, and this is so well accepted that it is mentioned in almost every article written about life-threatening fungal infection. Additionally, we analysed each fungal infection independently to avoid any bias or clouding of contrasting outcomes caused by different pathogenesis and the basic therapy regimen of fungal disease.

The meta-analysis of seven randomised controlled trials in HIV-infected patients with PCP found that the risk of death reduced to 62% (*p* = 0.001) in those treated with corticosteroids. Notably, the trials were in moderate and severe PCP patients, with a more substantial effect on worse disease. Although Montaner et al. [22, 83] had less severe patients in their studies, the effect of corticosteroids on survival could not be evaluated because of the crossover design of these two studies. There was insufficient evidence to conclude that supplementary corticosteroids affected mortality in babies with PCP and HIV (although it is likely). Evidence from RCTs for HIV-infected patients with mild PCP is still lacking. All these RCT studies were done in high-income countries with low *Mycobacterium tuberculosis* (TB) rates and were conducted 25 or more years ago. Other endemic infections in specific countries and populations may impact the value of corticosteroid treatment since uptake of highly active antiretroviral (HAART) and trimethoprim-sulfamethoxazole therapy is variable [84]. The value of corticosteroids for PCP in AIDS is of uncertain overall value in countries with high rates of tuberculosis.

*Aspergillus* spp., filamentous fungi, are frequently isolated from soil, organic matter, food, the indoor environment and hospitals [85, 86]. In our meta-analysis, all 11 cohort studies showed that steroid therapy had a worse outcome for both invasive aspergillosis and CPA. Notably, we found a major increase in death in IA of 250% (*p* < 0.001) in our pooled analysis and 270% in CPA. This is the first systematic review of this literature. Our review includes different underlying conditions linked with IA including chronic obstructive pulmonary disease (COPD) and coronavirus disease 2019 (COVID-19)-associated pulmonary aspergillosis (CAPA). All of them showed the same results in patients treated with steroids. Like our analysis, Raghu et al. presented a large cohort study from India (abstract only) and found that higher cumulative steroid dosage and longer duration of corticosteroid treatment are independent risk factors for both the development of CAPA and an increase in CAPA mortality [87]. Therefore, we recommend careful adherence to the existing steroid guidelines for COVID-19.

However, there are some limitations of our analysis of aspergillosis. First, all included studies were retrospective studies; some records may have bias and detailed laboratory records to confirm diagnoses were unavailable. In addition, our analysis of publication bias by funnel plot showed the possibility of bias (Supplementary data). The reason for this may because some small studies were not published. Moreover, researchers might prefer to report positive results showing higher mortality; negative or inconclusive studies might have been ignored. Although our analysis found that more than 2 mg per day per kg of steroid (prednisolone equivalents) use was a risk factor for death, this does not mean that less than 2 mg/kg/day of steroids is safe, due to lack of evidence.

*Candida* species are a significant source of hospital-acquired bloodstream infections and can cause serious infections linked with extended hospital stays and high fatality rates [88, 89]. Our meta-analysis showed that steroid treatment was a risk factor for mortality in candidemia and invasive candidiasis patients (213% increase, *p* < 0.001). However, there was no significant difference between steroid and non-steroid groups in the rare group with hepatosplenic candidiasis. Ours is the first study to integrate different observational studies of candidiasis with steroid treatment and outcome.

As for hepatosplenic candidiasis, Jang et al. [64] demonstrated no statistically significant differences in 90-day mortality between hepatosplenic candidiasis patients receiving corticosteroid medication and those who did not. Furthermore, Legrand et al. [54] showed that adjuvant corticosteroids reduced clinical symptoms and inflammatory responses in ten patients with hepatosplenic candidiasis, while Chaussade et al. reported rapid improvement in this disease’s symptoms in five patients with hepatosplenic candidiasis who were administered corticosteroids [90]. However, these earlier investigations did not identify the proportion of patients with hepatosplenic candidiasis who required corticosteroids due to incapacitating persistent fever, nor did they describe the clinical criteria that distinguished these patients from those who did not require corticosteroids. There is evidence that hepatosplenic candidiasis is a form of IRIS, and the corticosteroids or anti-inflammatory medications may benefit certain patients. Therefore, further studies should continue to explore the role of corticosteroid treatment in hepatosplenic candidiasis patients.

Long-term glucocorticoid use contributes to mucormycosis risk [91]. In our meta-analysis, we identified three studies which reported that corticosteroid treatment was a risk factor for death in patients with mucormycosis (increased risk of death of 419%, *p* = 0.001). Notably, mucormycosis pre-COVID-19 has a terrible prognosis, ranging from 33.3 to 80% overall mortality [92]. The limitation of our systematic review of mucormycosis is the small size of the three studies (total patients: 164) making it problematic to generalise our results, although the size of the effect is large. Additionally, two studies were related to COVID-19 patients while only one focused on other in-hospital patients, which may lead to a selection bias and not reflect the general effect of steroid treatment for mucormycosis outcome.

Fungal keratitis is challenging to identify and cure. In addition, it is frequently mistaken with other causes of infectious keratitis due to a lack of clinical and microbiological evidence in its early stages, resulting in delayed treatment. Very soon after corticosteroids were introduced in the 1950’s Thygeson warned that they might be significant factor in worsening fungal keratitis [93]. Since then, only one cohort study has addressed this concern and the published data indicate that recurrent overall treatment results were poorer in patients treated with topical steroids [76]. In contrast, topical corticosteroids after corneal grafting appear to be beneficial in preventing rejection. Topical corticosteroids are presently regarded as the optimum therapy for avoiding early immunological rejection and managing inflammation following keratoplasty [94, 95, 96].

Our analysis of topical corticosteroids and fungal keratitis has some limitations. First, only one study addressed the negative effect of steroid treatment in the early phases of fungal keratitis, while the diagnosis is being considered; and the other only addressed corneal graft rejection. The study in South Korea was conducted at a single tertiary hospital. Perhaps these findings cannot be generalised.

Rajasingham estimated a mean worldwide cryptococcal antigenaemia prevalence of 4% (95%CI: 16–74) among HIV-positive individuals with CD4 counts of fewer than 200 cells/L, equivalent to 179 000 (IQR 133 000–219 000) cases of cryptococcal antigenaemia globally in 2020. Annually, 152 000 cases (111 000–185,000) of cryptococcal meningitis were reported, resulting in 112 000 deaths attributable to cryptococcal disease (79 000–134 000). Cryptococcal illness is responsible for 19% (13–24) of AIDS-related deaths worldwide [97]. Our systematic review found three studies related to cryptococcal meningitis with steroid treatment, with contrasting results. A clinical cohort study demonstrated that steroid therapy dramatically decreased blindness in immunocompetent subjects with infection caused by *C. gattii* complex [73]. However, Beardsley et al. [74] found that steroids did not reduce mortality among patients with HIV-associated cryptococcal meningitis, but also were not harmful overall. Recently Anjum et al. [75] demonstrated that pulse corticosteroid therapy is associated with improved visual field outcome, and the patients they chose were previously healthy patients with the post-infectious inflammatory syndrome following cryptococcal meningitis, similar to Seaton’s study group. As in PCP, there appears to be a dichotomy between HIV and non-HIV infected patients: with a reversal of benefit in non-HIV. In non-HIV patients without immunosuppression, there is the likelihood of benefit. The main limitations of the data related to non-HIV patients are the study design, timing and dose of corticosteroids and relatively small number of patients. A randomised study could explore the dose of steroids in patients with cryptococcal meningitis without immunosuppression, although an adaptive design should be adopted to reduce the number of participants who are exposed to the inferior arm (whichever that is).

*Coccidioides immitis* and *C. posadasii* are sibling species of endemic fungi indigenous to the Americas, notably southwestern deserts of the USA and other arid areas in Mexico and further south [98]. Our systematic review focused on mortality, and we found two studies that reported the effect of corticosteroid treatment on the outcome of coccidioidomycosis. Blair’s [79] 16-year study comprised 55 patients, with the majority of infections (43/55, 78%) occurring in the lungs. According to the study, 34 patients used corticosteroids, and 16 individuals died. However, the number of deaths in the steroid group was not specified. They discovered that steroid use was associated with increased mortality along with hematologic malignancy (*p* = 0.02). On the other hand, Azadeh et al. identified 74 patients with primary pulmonary coccidioidomycosis [80]. They discovered that corticosteroid medication might assist immunocompetent patients with coccidioidomycosis in recovering faster. In addition, amphotericin B deoxycholate (AmBd) is used to treat coccidioidal meningitis but the majority of patients who receive intrathecal AmBd will have headache, nausea and vomiting [99]. Notably, concurrent intrathecal injection of corticosteroids, such as hydrocortisone or methylprednisolone, reduces the toxicity of the drug in the spinal canal, without apparently prejudicing outcome [100]. A previous review [101] concluded that long-term glucocorticoids might have deleterious effects on coccidioidomycosis patients. We found the opposite result in that steroid treatment may help to relieve symptoms faster in primary infection. However, it is difficult to conclude this, as the sample size is too small. As for bacterial meningitis, such as tuberculous meningitis, some researchers have found that adjunctive corticosteroids reduced death when compared with antibacterial treatment alone [102, 103]. Further study should extend the research time and increase the number of patients. Furthermore, corticosteroids’ influence on the clinical course of coccidioidomycosis, including coccidioidal meningitis, warrants more investigation.

*Fusarium species* cause a variety of human illnesses, including superficial, locally invasive and disseminated infections. Invasive fusariosis is a particular problem in neutropenic patients, with over 90 documented cases [104, 105, 106, 107]. In our meta-analysis, we identified one study which reported that corticosteroid therapy was a risk factor for disseminated fusariosis. The authors also revealed that corticosteroid therapy led to a poor outcome for patients with fusariosis. Regarding the clinicopathologic characteristics of fusariosis in individuals with hematologic disorders these data confirm and expand the findings of earlier large series [108]. Other studies have addressed the prognosis of fusariosis, but steroid therapy was not included as a mortality factor.

Disseminated histoplasmosis (DH) is a persistently progressive granulomatous illness caused by *Histoplasma capsulatum*, an intracellular dimorphic fungus [109]. Although we did not find any papers about the relationship between the mortality of DH and corticosteroids treatment, a previous case report and literature review found that a high dosage of corticosteroids was the most prominent factor for deadly infection [110]. In addition, a recent study found that corticosteroid use was an independent predictor of DH infection [111].

Our meta-analysis provides a comprehensive overview of the published data linking outcome and corticosteroid treatment in patients with different fungal diseases. However, it has several limitations. First, most studies had a retrospective cohort design, possibly with inadequate data on confounding factors. There may be a selection bias in the studied predictors, as different doctors collected the detail on each case, and missing data plagues such research efforts. Second, there were no natural control groups in some cohort studies as these studies addressed many aspects of the disease not only outcomes. Some biases in data selection and analysis are likely. Third, the dosage and timing of corticosteroid therapy were not included in most papers, except for aspergillosis. It is likely that both dose and duration are important in raising both the risk of fungal disease and affecting outcome. The current published data cannot address this meaningfully. This makes it difficult to advise clinicians on minimising adverse outcomes based on corticosteroid dosing. Fourth, our study did not include the effect of inhaled steroids in lung fungal infection because there has been little study of inhaled steroids and patient survival in complex clinical settings. Lastly, we found that some fungal diseases (allergic fungal rhinosinusitis, talaromyces and dimorphic fungal disease as examples) do not report any outcome of corticosteroid treatment. We did not include allergic bronchopulmonary aspergillosis in our analysis. We have not addressed the likely impact of azole/corticosteroid interactions, which may have acted to effectively boost corticosteroid exposure, in those taking prednisolone and voriconazole and methylprednisolone and itraconazole, as examples. Moreover, no study reported renal transplant loss related to withdrawal or not of corticosteroid therapy in the context of life-threatening fungal infection [112]. Future research could focus on the impact of corticosteroids on the outcome in renal transplant recipients and explore if reduction in steroid dose could have a positive impact on retaining renal allografts, without the patient dying.

## Conclusion

Our review demonstrates that with a few notable exceptions, corticosteroid therapy worsens outcome in several fungal diseases. The inter-relationship between corticosteroid dose, duration and outcome was more difficult to tease apart. Where the data is weak and somewhat equivocal, randomised or careful case-controlled studies should examine the impact of corticosteroid dose on the prognosis of various fungal infections.

## Supplementary Information

Below is the link to the electronic supplementary material.

Supplementary file1 (DOCX 364 KB)
