# Supplementary material for: The Impact of Corticosteroids on the Outcome of Fungal Disease: a Systematic Review and Meta-analysis
Source: Curr Fungal Infect Rep. 2023 Feb 23;17(1):54–70. doi: 10.1007/s12281-023-00456-2 (PMC9947451; doi:10.1007/s12281-023-00456-2)

**Li and Denning**

**Supplementary data**

**Table 1a.** Newcastle-Ottawa scores for Aspergillosis (cohort studies)

| Items | Selection |  |  |  | Comparability | Outcome |  |  | Total  scores |
| --- | --- | --- | --- | --- | --- | --- | --- | --- | --- |
|  | **Representativeness of the exposed cohort** | **Selection of the non-exposed cohort** | **Ascertainment of exposure** | **Outcome of interest was not present at start of study** |  | **Assessment of outcome** | **Long enough to assess outcomes** | **Adequacy of follow-up of cohorts** |  |
| Ribaud, 1999 | * |  | * | * | * | * | * |  | 6 |
| Fukuda, 2003 | * |  |  | * |  | * | * | * | 5 |
| Cordonnier, 2006 | * |  | * | * |  | * | * | * | 6 |
| Kiertiburanakul,  2007 | * |  | * | * |  | * | * |  | 5 |
| Upton,  2007 | * |  | * | * |  | * | * | * | 6 |
| Li,  2011 | * |  | * | * | * | * | * | * | 7 |
| Safdar,  2015 | * |  | * | * |  | * | * | * | 6 |
| Miceli,  2017 | * |  | * | * |  | * | * |  | 5 |
| Naito,  2018 | * |  | * | * | * | * | * |  | 6 |
| Gu,  2021 | * |  | * | * |  | * | * | * | 6 |
| Lee,  2022 | * |  | * | * | * | * | * | * | 7 |

**Table 1b.** Newcastle-Ottawa scores for Candidiasis and Candidemia (cohort studies)

| Items | Selection |  |  |  | Comparability | Outcome |  | |  | Total  scores |
| --- | --- | --- | --- | --- | --- | --- | --- | --- | --- | --- |
|  | **Representativeness of the exposed cohort** | **Selection of the non-exposed cohort** | **Ascertainment of exposure** | **outcome of interest was not present at start of study** |  | **Assessment of outcome** | **long enough for outcomes** | | **Adequacy of follow-up of cohorts** |  |
| Viudes, 2002 | * |  | * |  |  | * | * |  | | 4 |
| Chen, 2006 | * |  | * | * |  | * | * | | * | 6 |
| Labelle, 2008 | * |  | * | * |  | * | * | | * | 6 |
| Legrand, 2008 | * |  | * | * |  | * | * | | * | 6 |
| Neofytos, 2010 | * |  | * |  |  | * | * | | * | 5 |
| Slavin, 2010 | * |  | * | * |  | * | * | | * | 6 |
| Guimaraes, 2012 | * |  | * | * |  | * | * | | * | 6 |
| Santolaya, 2014 | * |  | * | * | * | * | * | | * | 7 |
| Colombo, 2014 | * |  | * | * |  | * | * | | * | 6 |
| Klingspor,2015 | * |  | * | * | * | * | * | | * | 7 |
| Kang, 2017 | * |  | * | * | * | * | * | |  | 6 |
| Ding, 2018 | * |  | * | * |  | * | * | | * | 6 |
| Jang, 2018 | * | * | * |  |  | * | * | | * | 6 |
| Alves, 2020 | * |  |  | * |  | * | * | | * | 5 |
| Chakrabarti, 2020 | * |  | * | * | * | * | * | | * | 7 |
| Kayaaslan, 2021 | * |  | * | * | * | * | * | | * | 7 |
| Boussen, 2022 | * |  | * | * |  | * | * | | * | 6 |

**Table 1b1.** Newcastle-Ottawa scores for Candidiasis and Candidemia (case control study)

| Items | Selection |  |  |  | Comparability | Outcome |  |  | Total  scores |
| --- | --- | --- | --- | --- | --- | --- | --- | --- | --- |
|  | **Case definition adequate** | **Representativeness of the cases** | **Selection of Controls** | **Definition of Controls** |  | **Ascertainment of exposure** | **Same method of ascertainment for cases and controls** | **Non-Response rate** |  |
| Munoz, 2011 | * | * |  | * | * | * | * |  | 6 |

**Table 1c.** Newcastle-Ottawa scores for *Pneumocystis jirovecii* pneumonia (cohort studies)

| Items | Selection |  |  |  | Comparability | Outcome |  |  | Total  scores |
| --- | --- | --- | --- | --- | --- | --- | --- | --- | --- |
|  | **Representativeness of the exposed cohort** | **Selection of the non-exposed cohort** | **Ascertainment of exposure** | **outcome of interest was not present at start of study** |  | **Assessment of outcome** | **long enough for outcomes** | **Adequacy of follow-up of cohorts** |  |
| Delclaux, 1999 | * |  | * | * | * | * | * | * | 7 |
| Pagano, 2002 | * |  | * |  | * | * | * | * | 6 |
| Zahar, 2002 | * |  | * |  | * | * | * | * | 6 |
| Roblot, 2003 | * |  | * | * | * | * | * |  | 6 |
| Bolle´e, 2007 | * |  | * |  | * | * | * |  | 5 |
| Moon, 2011 | * |  | * |  | * | * | * | * | 6 |
| Kofteridis, 2014 | * |  | * |  | * | * | * | * | 6 |
| Wieruszewski, 2018 | * |  | * | * | * | * | * | * | 7 |
| Liu, 2019 | * |  | * | * | * | * | * | * | 7 |
| Inoue, 2019 | * |  | * |  | * | * | * |  | 5 |
| Assal, 2021 | * |  | * | * | * | * | * | * | 7 |

**Table 1c1.** Newcastle-Ottawa scores for *Pneumocystis jirovecii* pneumonia (case control study)

| Items | Selection |  |  |  | Comparability | Outcome |  |  | Total  scores |
| --- | --- | --- | --- | --- | --- | --- | --- | --- | --- |
|  | **Case definition adequate** | **Representativeness of the cases** | **Selection of Controls** | **Definition of Controls** |  | **Ascertainment of exposure** | **Same method of ascertainment for cases and controls** | **Non-Response rate** |  |
| Pareja, 1998 | * | * |  | * | * | * | * |  | 6 |

**Table 1d.** Newcastle-Ottawa scores for fungal keratitis (cohort study)

**
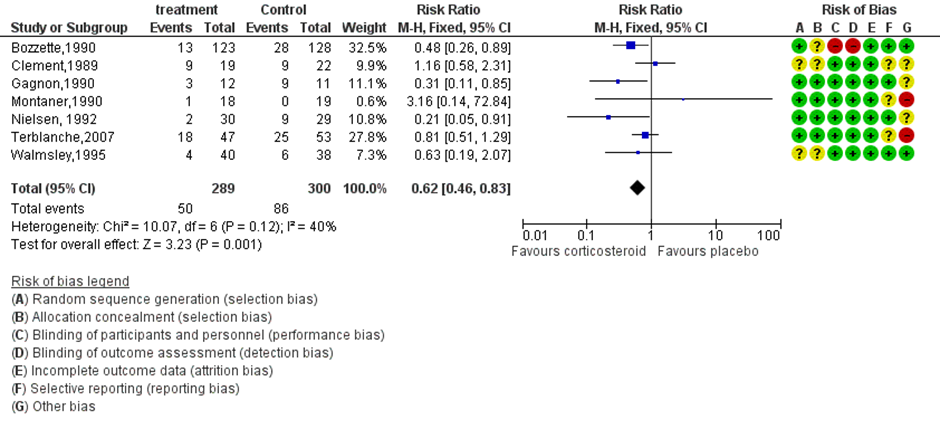
**

| Items | Selection |  |  |  | Comparability | Outcome |  |  | Total  scores |
| --- | --- | --- | --- | --- | --- | --- | --- | --- | --- |
|  | **Representativeness of the exposed cohort** | **Selection of the non-exposed cohort** | **Ascertainment of exposure** | **outcome of interest was not present at start of study** |  | **Assessment of outcome** | **long enough for outcomes** | **Adequacy of follow-up of cohorts** |  |
| Wang, 2016 | * |  | * | * |  | * | * | * | 6 |

**Table 1d1.** Newcastle-Ottawa scores for fungal keratitis (case control study)

| Items | Selection |  |  |  | Comparability | Outcome |  |  | Total  scores |
| --- | --- | --- | --- | --- | --- | --- | --- | --- | --- |
|  | **Case definition adequate** | **Representativeness of the cases** | **Selection of Controls** | **Definition of Controls** |  | **Ascertainment of exposure** | **Same method of ascertainment for cases and controls** | **Non-Response rate** |  |
| Cho, 2019 | * | * |  | * | * | * | * | * | 7 |

**Table 1e.** Newcastle-Ottawa scores for mucormycosis (cohort study)

| Items | Selection |  |  |  | Comparability | Outcome |  |  | Total  scores |
| --- | --- | --- | --- | --- | --- | --- | --- | --- | --- |
|  | **Representativeness of the exposed cohort** | **Selection of the non-exposed cohort** | **Ascertainment of exposure** | **outcome of interest was not present at start of study** |  | **Assessment of outcome** | **long enough for outcomes** | **Adequacy of follow-up of cohorts** |  |
| Kennedy, 2016 | * |  | * |  |  | * | * | * | 5 |
| Moorthy, 2021 | * |  | * | * | * | `* | * | * | 7 |

**Table 1e1.** Newcastle-Ottawa scores for mucormycosis (case control study)

| Items | Selection |  |  |  | Comparability | Outcome |  |  | Total  scores |
| --- | --- | --- | --- | --- | --- | --- | --- | --- | --- |
|  | **Case definition adequate** | **Representativeness of the cases** | **Selection of Controls** | **Definition of Controls** |  | **Ascertainment of exposure** | **Same method of ascertainment for cases and controls** | **Non-Response rate** |  |
| Choksi, 2022 | * | * | * | * | * | * | * | * | 8 |

**Table 1f.** Newcastle-Ottawa scores for cryptococcal meningoencephalitis (cohort study)

| Items | Selection |  |  |  | Comparability | Outcome |  |  | Total  scores |
| --- | --- | --- | --- | --- | --- | --- | --- | --- | --- |
|  | **Representativeness of the exposed cohort** | **Selection of the non-exposed cohort** | **Ascertainment of exposure** | **outcome of interest was not present at start of study** |  | **Assessment of outcome** | **long enough for outcomes** | **Adequacy of follow-up of cohorts** |  |
| Seaton, 1997 | * |  | * | * |  | `* | * | * | 6 |

**Table 1g.** Newcastle-Ottawa scores for coccidioidomycosis (cohort studies)

| Items | Selection |  |  |  | Comparability | Outcome |  |  | Total  scores |
| --- | --- | --- | --- | --- | --- | --- | --- | --- | --- |
|  | **Representativeness of the exposed cohort** | **Selection of the non-exposed cohort** | **Ascertainment of exposure** | **outcome of interest was not present at start of study** |  | **Assessment of outcome** | **long enough for outcomes** | **Adequacy of follow-up of cohorts** |  |
| Blair, 2005 | * |  | * |  |  | `* | * |  | 4 |
| Azadeh, 2013 | * |  | * |  |  | * | * | * | 5 |

**Table 1h.** Newcastle-Ottawa scores for fusariosis

| Items | Selection |  |  |  | Comparability | Outcome |  |  | Total  scores |
| --- | --- | --- | --- | --- | --- | --- | --- | --- | --- |
|  | **Representativeness of the exposed cohort** | **Selection of the non-exposed cohort** | **Ascertainment of exposure** | **outcome of interest was not present at start of study** |  | **Assessment of outcome** | **long enough for outcomes** | **Adequacy of follow-up of cohorts** |  |
| Nucci,2 003 | * |  | * | * | * | * | * | * | 7 |

**Publication bias assessment**

Publication bias is the failure to publish study results based on the direction or significance of the study's findings [1]. This may imply that only positive research with statistically significant results are published, while statistically insignificant or negative studies are not. A funnel plot is utilized by systematic reviews and meta-analyses to determine the existence of publication bias or systematic heterogeneity in the included research. If the plot has an inverted funnel shape that is symmetric, publication bias is improbable [2]. If the funnel plot is asymmetric, there is a systematic distinction between studies with higher and lower precision.

1. DeVito NJ, Goldacre B. Catalogue of bias: publication bias. BMJ Evid Based Med. 2019;24(2):53-4.

2. Nair AS. Publication bias - Importance of studies with negative results! Indian J Anaesth. 2019;63(6):505-7.

**S1.** Funnel plot of PCP : The Begg’s test for publication bias was not statistically

significant (P value = 0.586).


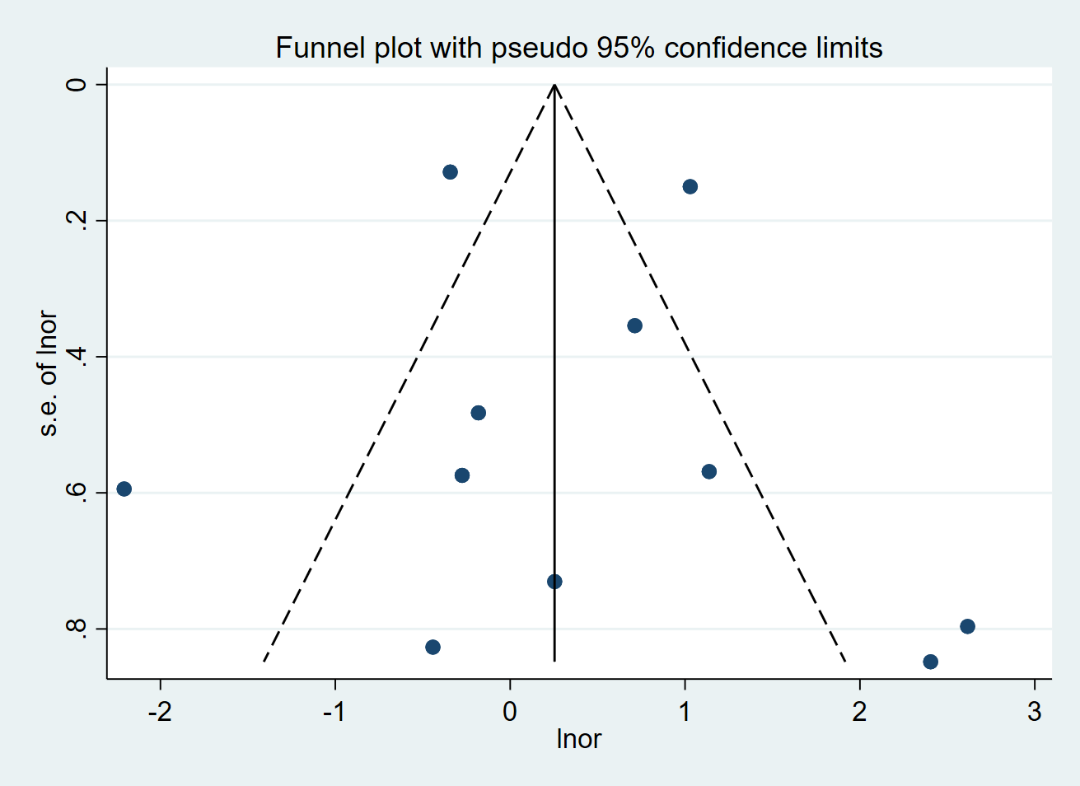


**S2.** Funnel plot of candidasis: The Begg’s test for publication bias was not statistically

significant (P value = 0.322).


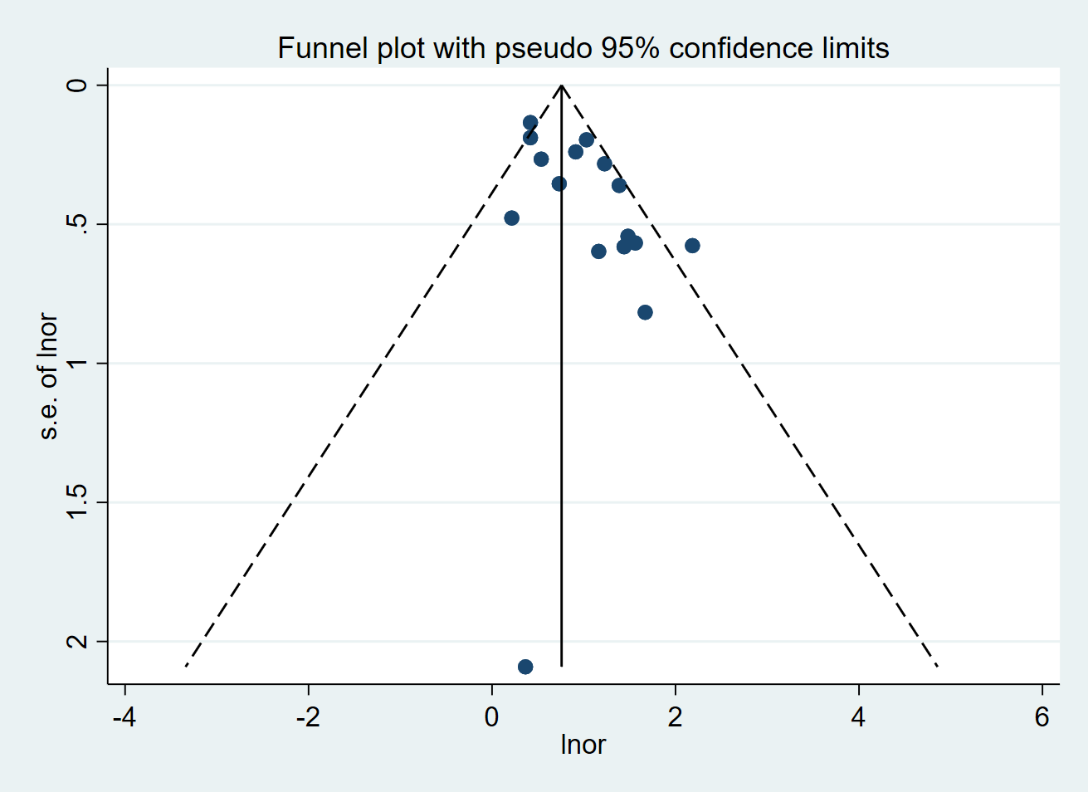


**S3.** Funnel plot of aspergillosis: The Begg’s test for publication bias was statistically

significant (P value = 0.024).


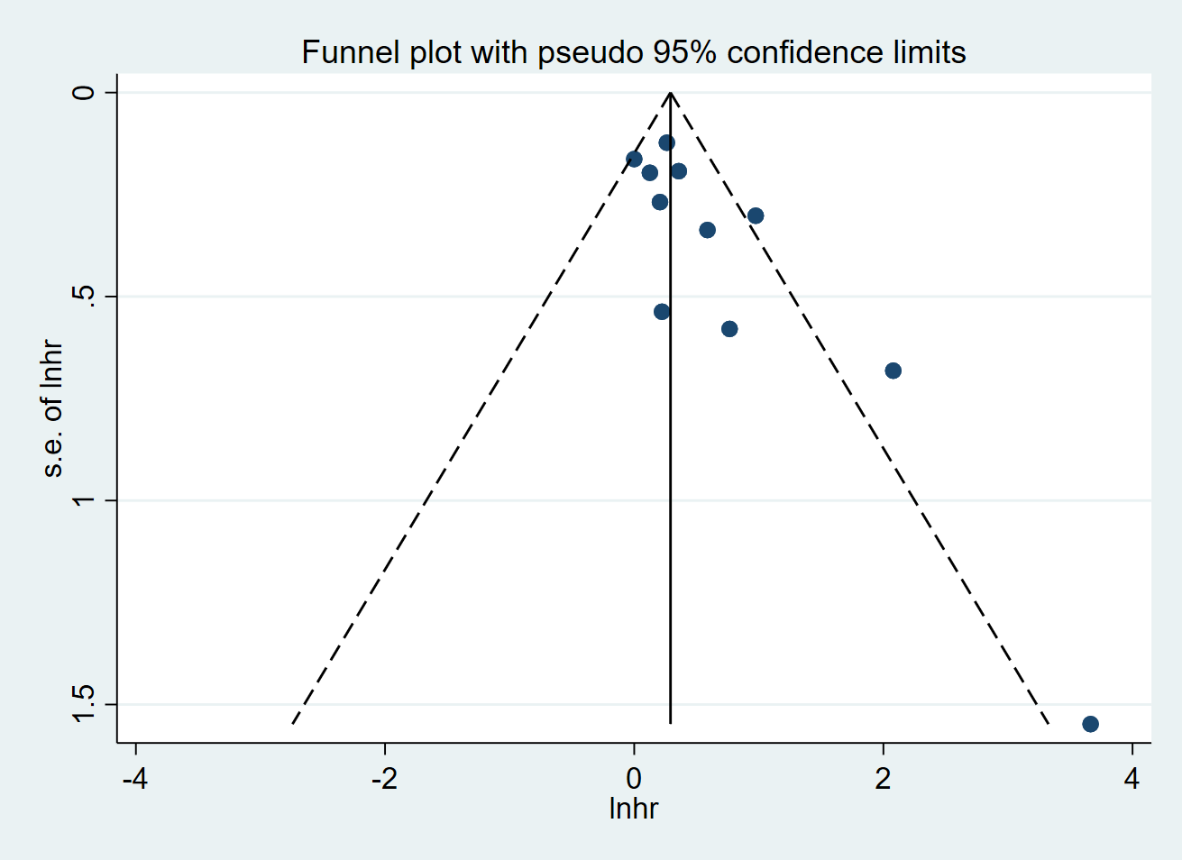

Supplement: Supplementary file 1 — Supplementary file1 (DOCX 364 KB) [file 12281_2023_456_MOESM1_ESM.docx]
